# Omics analysis of the effect of cold normal saline stress through gastric gavage on LPS induced mice

**DOI:** 10.3389/fmicb.2023.1256748

**Published:** 2023-12-14

**Authors:** Jing Li, Zhihao Cui, Ming Wei, Mikhlid H. Almutairi, Peishi Yan

**Affiliations:** ^1^College of Animal Science and Technology, Nanjing Agricultural University, Nanjing, China; ^2^Department of Zoology, College of Science, King Saud University, Riyadh, Saudi Arabia

**Keywords:** cold stress, LPS, mice, microbiome, metabolism

## Abstract

Cold stress is a significant environmental stimulus that negatively affects the health, production, and welfare of animals and birds. However, the specific effects of cold stimulation combined with lipopolysaccharide (LPS) on the mouse intestine remain poorly understood. Therefore, we designed this research to explore the effect of cold stimulation + LPS on mice intestine via microbiome and microbiota sequencing. Forty-eight mice were randomly divided into four experimental groups (*n* = 12): Control (CC), LPS-induced (CL), cold normal saline-induced (MC) and LPS + cold normal saline-induced (ML). Our results showed body weight was similar among different groups of mice. However, the body weight of mice in groups CC and CL were slightly higher compared to those in groups MC and ML. The results of gene expressions reflected that CL and ML exposure caused gut injury and barrier dysfunction, as evident by decreased *ZO-1*, *OCCLUDIN* (*P* < 0.01), and *CASPASE-1* (*P* < 0.01) expression in the intestine of mice. Moreover, we found that cold stress induced oxidative stress in LPS-challenged mice by increasing malondialdehyde (MDA) accumulation and decreasing the antioxidant capacity [glutathione peroxidase (GSH-Px), superoxide dismutase (SOD), total and antioxidant capacity (T-AOC)]. The cold stress promoted inflammatory response by increased IL-1β in mice treated with cold normal saline + LPS. Whereas, microbiome sequencing revealed differential abundance in four phyla and 24 genera among the mouse groups. Metabolism analysis demonstrated the presence of 4,320 metabolites in mice, with 43 up-regulated and 19 down-regulated in CC vs. MC animals, as well as 1,046 up-regulated and 428 down-regulated in ML vs. CL animals. It is Concluded that cold stress enhances intestinal damage by disrupting the balance of gut microbiota and metabolites, while our findings contribute in improving management practices of livestock in during cold seasons.

## Introduction

Cold stress is an important environmental stimulation factor to animals and human beings in cold regions and during wintertime in other regions, which bring negative effects on health, production and welfare of animals and birds (Wei et al., 2018; Liu et al., 2022). Previous studies found that cold stimulation effect the productivity, oxidative resistance and immune dysfunction (Li et al., 2020; Liu et al., 2022). The enteric canal is a useful organ for nutrient absorption and regulation of immune function (Lee et al., 2018), and this organ is sensitive to stressors like cold stimulation, which cause inflammation reactions, oxidative stress, and intestinal injury in animals (Fu et al., 2013; Zhao et al., 2013). Many intestinal pathogens are related to stress like inflammatory bowel disease and injury (Lee et al., 2018). Water is important for body health and physiological activities, but it is reported that weaned piglets drinking warm (30°C) water has a better feed-to-weight ratio than those drinking cold (13°C) water (Zhang et al., 2020), which implied that low-temperature drinking water is a cold stress factor. Lipopolysaccharide (LPS) is a pathogenic component derived from gram-negative bacteria, which produce an immune response and lead to injury through oxidative damage (Meng et al., 2022). LPS is widely used to induce enteric canal inflammation and oxidative damage in different animals (Hassan et al., 2021; Feng et al., 2023).

Gut microbiota comprises of trillions of microorganisms, such as archaea, parasites, fungi, viruses, and bacteria (Naibaho et al., 2021; Ruigrok et al., 2023). These microorganisms contribute to the absorption and metabolism of nutrition, protect against pathogens, and is helpful to develop host’s immune system (Nishida et al., 2018). Gut dysbiosis is commonly linked with intestinal diseases like irritable bowel syndrome (Nishino et al., 2018), inflammatory bowel disease (Matsuoka and Kanai, 2015), and salmonellosis (Aljahdali et al., 2020). Previous studies have confirmed an intestinal imbalance in LPS-induced animals (Chen et al., 2022; Ruan et al., 2022; Wang et al., 2022).

Lipopolysaccharide is known as a major activator of the inflammatory response, it binds to toll-like receptor 4 (TLR4), activates nuclear factor kappa B (NF-κB) and enhances the inflammation through the production of pro-inflammatory cytokines and injury to endothelial cells (Joo et al., 2016). In rat and rabbit animal models, LPS-induced systemic inflammation is depend on several factors including ambient temperature and LPS dose (Romanovsky et al., 2005). At a low temperature (cold stress), low doses of LPS causes fever and several sequential, while at neutral temperature even high doses of LPS cause low fever and less detrimental effects (Romanovsky et al., 2005; Rudaya et al., 2005).

Thus, we hypothesized that exposure to cold temperature is a factor that aggravates inflammation. To evaluate this hypothesis, we investigated the effect of cold stress on the severity of inflammatory responses due to LPS in mouse model. Therefore, we examined the impact of cold stress and LPS on the mouse intestine via microbiome and microbiota sequencing.

## Materials and methods

### Animals, experimental design and sample collection

**FIGURE 1:**
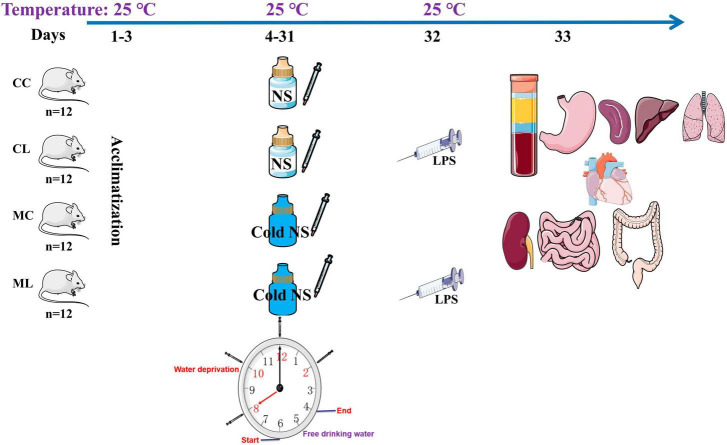
Research design diagram of the present experiment.

A total of 48 four-week-old ICR mice (24 males and 24 females) with a middle weight of 18 ± 2.2 g was purchased from Qinglongshan Animal Breeding (Nanjing, China). After 3-day of acclimatization period, mice were randomly divided into four groups: control group (CC), LPS-induced group (CL), cold normal saline-induced group (MC), and LPS + cold normal saline-induced group (ML) as shown in Figure 1. Mice in group CC and CL were administered room temperature normal saline (25°C) by gavage from day 4th to 31st, while LPS was administered only in CL group on 32nd day. Whereas, mice in group MC and ML were administered cold normal saline (4°C) at a dosage of 0.5 mL per mouse every 2 h for four times daily to induce cold stress from day 4th to 31st, while LPS was administered only in ML group on 32nd day. On day 32nd, mice in groups CL and ML were infected with 20 mg/kg LPS (Solarbio life science, China) according to previous study (Chen et al., 2022). All the groups were kept and reared on same ambient temperature at 25°C throughout the experimental period from day 1st to 32nd. After 1-day of LPS administration on 33rd day, the mice in all the groups were euthanized to collect serum, heart, liver, kidney, lung, spleen, stomach, jejunum, ileum, cecum, colon, and rectum. The mice were provided the Pellet diet and water *ad libitum* throughout the experimental, and daily body weights and diarrhea were also recorded.

### Hematoxylin and eosin staining

Tissue samples including the spleen, stomach, jejunum, ileum, cecum, and colon, were collected from mice of all the groups and fixed in 4% paraformaldehyde for 24 h and subjected to H&E staining. The tissues fixed in the formalin were processed further to observe the histopathological lesions by following the routinely used procedures like dehydrations, embedding, sectioning, mounting and staining. Thick sections of the tissues about 4–5 μm were cut and stained by the Hematoxylin and Eosin staining techniques. The histological sections were examined using a CX23 microscope (Olympus Co., Tokyo, Japan). The villus height and crypt depth of each selected mouse were measured following the methods described by Chen et al. (2022).

### Antioxidant indexes, NO, and cytokine levels examination

The serums obtained from mice were kept at −20°C for further assays. For the antioxidant capacity indexes, superoxide dismutase, glutathione peroxidase, total anti-oxidation capacity, NO, and malondialdehyde were measured using commercially available kits following the manufacturer’s instructions (Nanjing Jiancheng Bioengineering Research Institute Co., Ltd., China). Tumor necrosis factor (TNF-α), interleukin 1 beta (IL-1β), interleukin-6 (IL-6) and IL-10 were detected in the blood serum of mice through specific kits (Solarbio life science, China).

### Gut microbiome analysis

Mice rectums of all the groups (CC, CL, MC, and ML) were used to extract genomic DNA (gDNA) by employing the GenElute™ Microbiome DNA Purification Kit (Sigma-Aldrich, Germany), following the manufacturers instructions. The concentration and integrity of the DNA products were surveyed via NanoDrop 2000 spectrophotometer (Thermo Scientific, USA) and agarose gel electrophoresis. The targeting regions of the microbial 16S rRNA (V3-V4) gene were amplified using the forward primer 338F (5′-ACTCCTACGGGAGGCAGCAG-3′) and the reverse primer 806R (5′-GGACTACHVGGGTWTCTAAT-3′). Subsequently, amplicon sequencing of the ICR animals was conducted using the Illumina platform at Bioyi Biotechnology Co., Ltd., as described in previous studies (Chen et al., 2022; Dong et al., 2023). Following sequencing, Trimmomatic, Cutadapt, QIIME2, and DADA2 were utilized to generate accurate and reliable data for subsequent bioinformatic analysis (Edgar, 2013; Callahan et al., 2016; Bolyen et al., 2019). High-quality sequences with a similarity threshold of 97% were clustered into operational taxonomic units (OTUs) using USEARCH (Edgar, 2013) and assigned taxonomic annotations by aligning them with the SILVA database (Bolyen et al., 2019). A Venn map was constructed to identify the shared OTUs among the groups following the previous method (Chen and Boutros, 2011). The annotation of the microbial communities was visually displayed using KRONA software as outlined by Ondov et al. (2011). Alpha diversity metrics, including Chao1, Ace, Shannon, Simpson, and PD_whole_tree, were calculated to assess the individual microbial diversity. Beta diversity analysis, including Principal Component Analysis, Principal Coordinates Analysis, Non-Metric Multi-Dimensional Scaling, Unweighted Pair-group Method with Arithmetic Mean, and heat maps were performed to examine the variation in microbial communities across samples. These analyses were carried out using QIIME2 and R software as described by Bolyen et al. (2019). To uncover distinctive bacteria among the groups, we utilized various statistical methods and tools including analysis of variance, Wilcoxon rank-sum test, ternary phase diagram, Linear discriminant analysis Effect Size, Metastats, and statistical analysis of Metagenomic Profiles (White et al., 2009; Segata et al., 2011; Parks et al., 2014). Network analysis was performed using R to explore potential correlations among bacterial taxa. Additionally, the prediction of microbiota functional potential was conducted using PICRUSt2, targeting the Kyoto Encyclopedia of Genes and Genomes and Cluster of Orthologous Groups databases (Kanehisa and Goto, 2000; Langille et al., 2013).

### Metabolomics analysis

Metabolites from rectum samples (*n* = 6) of each group were extracted and subjected to metabolomics analysis via LC/MS (Dunn et al., 2011; Wang et al., 2016). Raw data processing and annotation were performed using MassLynx and Progenesis QI software (Wang et al., 2016). Spearman rank correlation and PCA were conducted to ensure the validity of current results. The annotation of metabolites was carried out using the KEGG, HMDB, and Lipidmaps databases (Fahy et al., 2007; Wishart et al., 2018).

Venn diagrams, PCA, and OPLS-DA were performed to investigate the variation between and within the groups (Chen et al., 2020). Remarkable differences in metabolomics among the mice groups were identified based on the variable importance in projection (VIP) values (>1) combined with statistical significance (*P* < 0.05). The differential metabolomics was described using multiple methods, including bar charts illustrating fold differences, volcano plots, cluster heatmaps, correlation graphs, z-score diagrams, radar charts and violin plots.

### qRT-PCR analysis

RNA extraction was performed from jejunum and ileum tissues of all the animals using Trizol reagent (Life Technologies, USA), and then the quality and quantity of RNA products were inspected via gel electrophoresis and Nanodrop 2000 (Thermo Fisher Scientific, China). The cDNA synthesis was carried out using Invitrogen™ kits (Thermo Fisher Scientific, USA), followed by RT-PCR analysis using 2X SYBR Green Fast qPCR Mix (ABclonal, China). The analysis was conducted using the StepOnePlus™ RT-PCR System (Applied Biosystems, USA). Three independently repeated reactions were performed for each mouse sample, and the relative quantification of genes was determined by using 2^–ΔΔCT^ method. The primers information is shown in Table 1.

**TABLE 1:** Primers used in the present study.

Genes	Primer sequence	Product size (bp)	Tm (°C)
Occludin	F: 5′–TGCTTCATCGCTTCCTTAGTAA–3′ R: 5′–GGGTTCACTCCCATTATGTACA–3′	155	54
*ZO-1*	F:5′– CTGGTGAAGTCTCGGAAAAATG–3′ R: 5′–CATCTCTTGCTGCCAAACTATC–3′	97	54
*NLRP3*	F: 5′–CATCAATGCTGCTTCGACAT–3′ R: 5′–TCAGTCCCACACACAGCAAT–3′	118	56
*CLAUDIN*	F: 5′–AGATACAGTGCAAAGTCTTCGA–3′ R:5′– CAGGATGCCAATTACCATCAAG–3′	86	54
*CASPASE-1*	F: 5′–TGCCCTCATTATCTGCAACA–3′ R: 5′–GATCCTCCAGCAGCAACTTC–3′	95	56
*B -ACTIN*	F:5′– CTACCTCATGAAGATCCTGACC–3′ R: 5′–CACAGCTTCTCTTTGATGTCAC–3′	90	54

### Statistical analysis

Analysis of variance (ANOVA) and Student’s *t*-test were employed to analyze the data. The statistical analysis was conducted using IBM SPSS software (version 26.0). The data are presented as means ± standard deviation (SD), and *P* < 0.05 was considered statistically significant.

## Results

### The effects of LPS on mice body weights, organ indexes, and intestines damage

**FIGURE 2:**
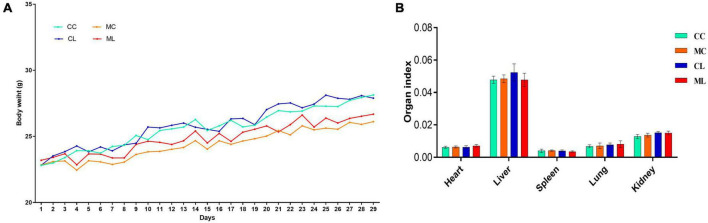
Effects of LPS on mice body weights **(A)** and organ indexes **(B)**. Scale bar 50 μm. Data were presented as the mean ± SEM (*n* = 3).

Similar body weight was observed in mice among all the experimental groups. However, the mice in groups CC and CL had slightly higher body weights compared to the mice in groups MC and ML (Figure 2A) but the difference is not significant (*P* > 0.05). Similarly, there was no prominent difference (*P* > 0.05) in the organ index between mouse groups (Figure 2B). Histopathological analysis revealed that LPS administration in groups CL and ML severely damaged the integrity of intestinal villi and gastric epithelium. The villus length was obviously shorter (*p* < 0.05) and crypt depth was observably longer (*p* < 0.05) in these mice, especially in animals in ML (Supplementary Figure 1).

**FIGURE 3:**
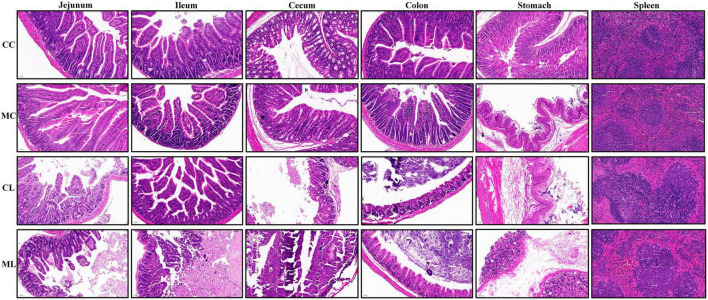
LPS caused damages in intestines, stomach and spleen in mice.

Additionally, the spleens of LPS-induced mice showed enlarged red pulps and increased leukomonocytes (Figure 3).

### The effects of cold normal saline stress on LPS induced mice on antioxidant indexes, NO and cytokine levels in serum

**FIGURE 4:**
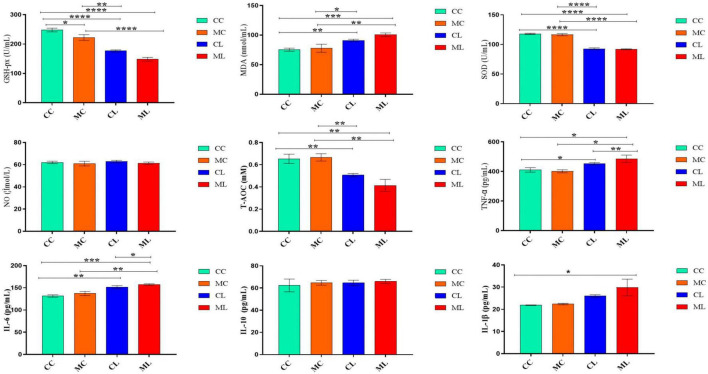
The effects of LPS on antioxidant indexes, NO and cytokine levels in serum. Significance is presented as *p < 0.05, **p < 0.01, ***p < 0.001, and ****p < 0.0001; data are presented as the mean ± SEM (*n* = 6).

The antioxidant indexes, including T-AOC, GSH-Px, and SOD were significantly (*P* < 0.05) lower in mice challenged with LPS and prolonged cold stress exposure compared with other groups. Conversely, the MDA level was markedly higher (*P* < 0.01) in mice particularly in the group treated with cold normal saline + LPS. Whereas, the level of nitric oxide (NO) and interleukin-10 (IL-10) were examined in different groups but the differences were non-significant (*P* > 0.05) in all the groups. However, the cytokines TNF-α and IL-6 (*P* < 0.01) were expressed higher significantly (*P* < 0.05) in mice challenged with LPS and prolonged cold stress exposure compared with other groups. Furthermore, IL-1β level was similar between groups CC and CL, but it was significantly (*P* < 0.05) elevated in mice treated with cold normal saline + LPS (Figure 4).

### The effects of cold normal saline stress on LPS induced mice on related genes’ expressions

**FIGURE 5:**
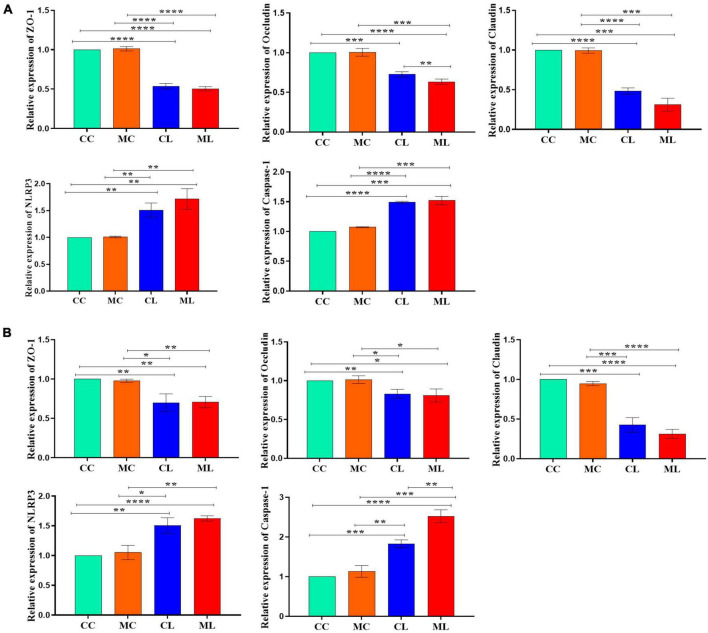
Relative expression analysis of ZO-1, Occludin, Claudin, Caspase-1 and NLRP3 via qRT-PCR, **(A)** jejunum, **(B)** ileum. Significance is presented as *p < 0.05, **p < 0.01, ***p < 0.001, and ****p < 0.0001; data are presented as the mean ± SEM (*n* = 3).

The expression levels of *ZO-1*, *OCCLUDIN*, and *CLAUDIN* in the jejunum were significantly (*P* < 0.05) decreased in mice challenged with LPS and cold stress as compared to other groups. Conversely, the expression levels of *CASPASE-1* and *NLRP3* were significantly increased (*P* < 0.05) in CL and ML groups compared with CC and MC groups (Figure 5A). Similar results were observed in the ileum in which *ZO-1*, *OCCLUDIN* and *CLAUDIN* expressions were significantly (*P* < 0.05) decreased in CL and ML groups compared with CC and MC groups. Whereas, the expression levels of *CASPASE-1* and *NLRP3* were significantly increased (*P* < 0.05) in mice challenged with LPS and prolonged cold stress exposure as compared to CC and MC groups (Figure 5B).

### The effects of LPS on the structure and diversity of animal gut microbiota

**FIGURE 6:**
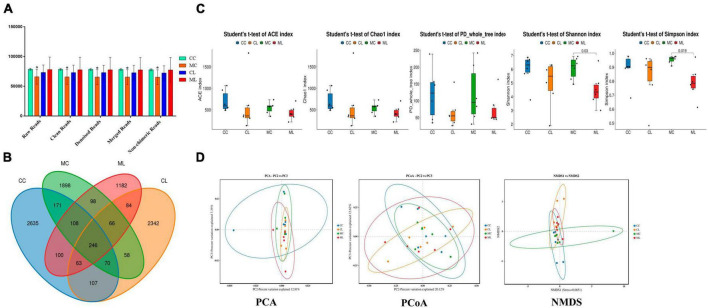
Effects of LPS on the structure and diversity of animal gut microbiota. **(A)** Sequencing data statistical analysis, **(B)** Length distribution of sequencing data, **(C)** Rank abundance curve, **(D)** Alpha diversity index analysis. Significance is presented as *p < 0.05, **p < 0.01, ***p < 0.001, and ****p < 0.0001; data are presented as the mean ± SEM (*n* = 6).

A total of 1,781,514 and 1,775,898 raw and clean reads, respectively were obtained from the current mice samples. Each group had more than 75,339, 48,262, 49,246, and 60,260 non-chimeric reads (Table 2). The number of data reads in group MC was noticeably (*P* < 0.05) lower compared to the CC group (Figure 6A). In total, 9,228 operational taxonomic units (OTUs) were identified in the mice, with 246 OTUs shared among the groups. Group CL shared 434 to 486 OTUs, while group ML shared 459 to 518 OTUs with the other groups (Figure 6B). Alpha diversity analysis indicated that Shannon (*P* < 0.05) and Simpson (*P* < 0.05) indices in ML were memorably lower than those in MC (Table 3; Figure 6C). Beta diversity analysis showed that the samples in groups MC, CL, and ML clustered closely together on PCA. The distance between groups CC and MC was short based on PCoA, while groups CL and ML were close to each other based on NMDS (Figure 6D).

**TABLE 2:** Statistical analysis of mouse samples sequencing data.

Sample ID	Raw reads	Clean reads	Denoised reads	Merged reads	Non-chimeric reads
CC1	76,318	76,123	75,965	75,831	75,339
CC2	79,710	79,546	79,475	79,303	78,613
CC3	79,832	79,616	79,578	79,366	79,162
CC4	77,558	77,319	77,215	77,014	76,930
CC5	79,901	79,684	79,532	79,277	79,101
CC6	80,020	79,836	79,786	79,539	79,337
CL1	79,896	79,677	79,665	79,642	79,598
CL2	79,979	79,759	79,562	79,207	78,628
CL3	79,762	79,572	79,442	78,964	77,710
CL4	78,135	77,868	77,722	77,692	77,198
CL5	48,593	48,397	48,317	48,283	48,262
CL6	75,015	74,752	74,608	74,581	74,351
MC1	69,957	69,784	69,717	69,510	69,293
MC2	49,502	49,343	49,332	49,248	49,246
MC3	79,983	79,724	79,614	79,486	79,179
MC4	60,134	59,844	59,775	59,576	58,688
MC5	57,577	57,410	57,218	57,083	56,915
MC6	79,811	79,609	79,548	79,459	79,417
ML1	68,956	68,759	68,704	68,659	68,575
ML2	80,034	79,783	79,615	79,425	79,139
ML3	69,063	68,761	68,676	68,581	68,439
ML4	71,884	71,593	71,344	71,308	71,240
ML5	60,568	60,321	60,288	60,277	60,260
ML6	119,326	118,818	118,780	118,552	118,308

**TABLE 3:** Statistical analysis of Alpha diversity index.

Sample	Feature	ACE	Chao1	Simpson	Shannon	PD_whole_tree	Coverage
CC1	627	629.1382	627.4912	0.9033	6.0561	99.0368	0.9999
CC2	485	486.3673	485.0938	0.9778	6.576	44.2971	0.9999
CC3	547	553.6486	549.7755	0.6798	3.7706	146.8667	0.9998
CC4	955	959.9665	955.6791	0.8934	5.6838	238.9361	0.9998
CC5	1061	1064.7711	1061.7258	0.9034	6.7134	157.1504	0.9999
CC6	517	520.0825	517.56	0.9772	6.8684	34.4298	0.9999
CL1	127	131.8703	128.2	0.4822	1.8885	26.0812	0.9999
CL2	1822	1823.9508	1822.1186	0.8838	6.2684	155.4129	0.9999
CL3	601	602.635	601.0971	0.9508	6.315	35.2559	0.9999
CL4	375	377.0643	375.5	0.9611	5.9666	54.0704	0.9999
CL5	304	304.5632	304.0435	0.7741	4.2534	72.4377	1
CL6	339	339.9516	339.375	0.9157	5.0324	56.0789	0.9999
MC1	589	591.3971	589.5385	0.9733	6.7646	106.8593	0.9999
MC2	350	350.6995	350.1765	0.9668	6.0784	53.4059	0.9999
MC3	728	736.0014	729.6132	0.9773	6.9336	242.2064	0.9998
MC4	439	439.3646	439	0.9374	5.2844	30.5536	1
MC5	594	595.7274	594.4773	0.9601	6.4219	83.3632	0.9999
MC6	569	571.886	569.3218	0.9094	4.9192	205.6026	0.9999
ML1	406	408.5056	406.6222	0.7965	4.1665	87.3503	0.9999
ML2	514	514.5357	514.0323	0.9729	6.6193	49.4554	1
ML3	393	393.525	393.0357	0.7429	4.5134	48.9056	1
ML4	332	333.5886	332.2857	0.8489	4.9739	44.7464	0.9999
ML5	216	217.5451	217.1538	0.7749	3.8148	48.7069	0.9999
ML6	705	708.6438	707.0192	0.6129	3.0064	163.7069	0.9999

### The effect of LPS on intestinal microbiota in different taxa

**FIGURE 7:**
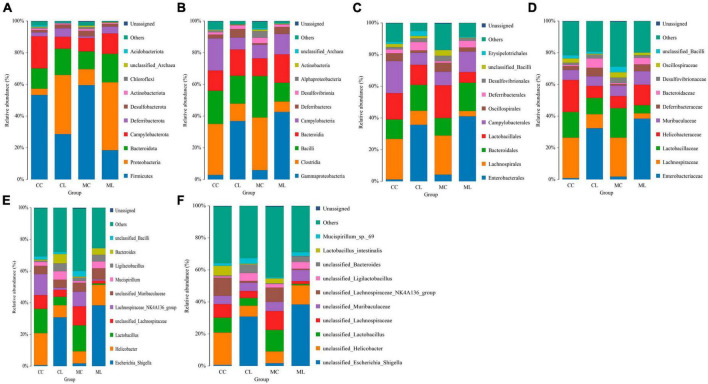
The effect of LPS on intestine microbiota in different taxa. **(A)** Phylum, **(B)** Class, **(C)** Order, **(D)** Family, **(E)** Genus, and **(F)** Species.

**FIGURE 8:**
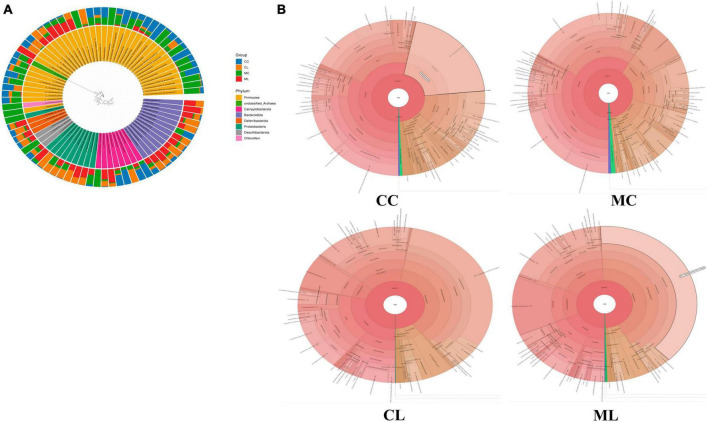
Microbiota composition analysis of mouse gut microbiota. **(A)** Phylogenetic tree distribution map, **(B)** Krona species annotation diagram.

At the phylum level, the ruling phyla in CC mice were Firmicutes (53.53%), Campylobacterota (20.01%), and Bacteroidota (12.91%), the ruling phyla in MC mice were Firmicutes (59.70%), Bacteroidota (11.6%) and Proteobacteria (9.96%), in CL animals were Proteobacteria (38.02%), Firmicutes (27.58%) and Bacteroidota (16.41%), while ruling phyla in ML animals were Proteobacteria (40.05%), Bacteroidota (19.40%) and Firmicutes (18.35%) (Figure 7A and Table 4). At the class level, the top three most abundant classes were Clostridia (32.23%), Bacilli (20.90%) and Campylobacteria (20.24%) in group CC, Gammaproteobacteria (36.90%), Bacilli (17.59%) and Bacteroidia (16.57%) in group CL, Clostridia (33.18%), Bacilli (26.2%) and Bacteroidia (11.19%) in group MC, while Gamma proteobacteria (42.66%), Bacteroidia (17.52%) and Campylobacteria (12.9%) in group ML (Figure 7B). At the order level, the main orders in group CC were Lachnospirales (25.56%), Campylobacterales (20.24%) and Lactobacillales (16.66%), in group CL were Enterobacterales (35.71%), Bacteroidales (16.32%) and Lactobacillales (12.73%), in group MC were Lachnospirales (24.62%), Lactobacillales (20.68%) and Bacteroidales (11.03%), and in group ML were Enterobacterales (41.02%), Bacteroidales (17.84%) and Campylobacterales (12.90%) (Figure 7C). At the family level, Lachnospiraceae (25.56%), Helicobacteraceae (20.21%) and Lactobacillaceae (16.19%) were mainly found in CC animals, Enterobacteriaceae (32.39%), Lactobacillaceae (10.28%) and Lachnospiraceae (8.78%) were primary families in CL mice, Lachnospiraceae (24.61%), Lactobacillaceae (18.61%) and Helicobacteraceae (7.66%) were mainly detected in MC animals, and Enterobacteriaceae (38.44%), Helicobacteraceae (12.90%) and Lactobacillaceae (5.12%) were principally examined in ML mice (Figure 7D). At the genus level, the staple genera were *Helicobacter* (20.21%), *Lactobacillus* (15.34%) and Lachnospiraceae_NK4A136_group (13.30%) in CC mice, *Escherichia_Shigella* (30.88%), *Helicobacter* (7.60%) and Bacteroides (5.91%) in CL animals, *Lactobacillus* (16.39%), unclassified_Lachnospiraceae (12.04%) and Lachnospiraceae_NK4A136_group (9.19%) in MC mice, *Escherichia_Shigella* (38.39%), *Helicobacter* (12.90%) and unclassified_Muribaculaceae (6.97%) in CL animals (Figure 7E). At species level, unclassified_*Helicobacter* (20.21%), unclassified_Lachnospiraceae_NK4A136_group (11.35%) and unclassified_*Lactobacillus* (9.30%) were mainly uncovered in mice in CC group, unclassified_*Escherichia_Shigella* (30.88%), unclassified_*Helicobacter* (6.75%) and unclassified_Bacteroides (5.28%) were revealed in mice in CL group, unclassified_*Lactobacillus* (13.38%), unclassified_Lachnospiraceae (11.82%) and unclassified_Lachnospiraceae_NK4A136_group (8.97%) were tested in mice in MC group, while unclassified_*Escherichia_Shigella* (38.39%), unclassified_*Helicobacter* (12.05%) and unclassified_Muribaculaceae (6.97%) were examined in mice in group ML (Figure 7F). Phylogenetic tree distribution analysis to top 80 abundant OTUs found that the abundance of g__Lachnospiraceae_NK4A136_group (ASV39), s__uncultured_*Clostridiales_bacterium* (ASV29), g__Lachnospiraceae_NK4A136_group (ASV9), g__Lachnospiraceae_NK4A136_group (ASV75), f__Lachnospiraceae (ASV52), s__Lachnospiraceae_bacterium_ DW59 (ASV77), g__*Roseburia* (ASV57), g__*Anaerotruncus* (ASV33), g__*Candidatus_Arthromitus* (ASV56), s__*Lactobacillus*_*intestinalis* (ASV7), g__*Helicobacter* (ASV5), g__*Helicobacter* (ASV38), g__*Helicobacter* (ASV24) and *Alloprevotella* (ASV36) decreased, especially in LPS induced mice, while g__*Enterococcus* (ASV67), g__Ligi*lactobacillus* (ASV6), g__Ligi*lactobacillus* (ASV64), s__*Malacoplasma_muris* (ASV18), s__*Mucispirillum*_sp._69 (ASV14), g__*Mucispirillum* (ASV12), g__Rodentibacter (ASV17), g__*Escherichia_Shigella* (ASV22), g__*Escherichia_Shigella* (ASV23), g__*Escherichia_Shigella* (ASV1), g__*Helicobacter* (ASV4), s__*Helicobacter*_*ganmani* (ASV40), g__*Parabacteroides* (ASV26), f__Muribaculaceae (ASV42), f__Muribaculaceae (ASV70), f__Muribaculaceae (ASV61), f__Muribaculaceae (ASV43), g__Bacteroides (ASV20), g__Bacteroides (ASV30), g__Bacteroides (ASV63), g__Bacteroides (ASV41) and g__Bacteroides (ASV74) increased in LPS challenged animals (Figure 8A). Krona species annotation showed that the main genera were unclassified__*Helicobacter* (20%), unclassified__Lachnospiraceae_NK4A136_group (11%), unclassified__Lachnospiraceae (9%) and unclassified__*Lactobacillus* (9%) in CC mice, unclassified__*Escherichia_Shigella* (31%), unclassified__*Helicobacter* (7%), unclassified__Bacteroides (5%), unclassified__Muribaculaceae (5%), unclassified__Ligi*lactobacillus* (5%) and unclassified__*Lactobacillus* (5%) in mice in CL, and unclassified__*Escherichia_Shigella* (38%), unclassified__*Helicobacter* (12%) and unclassified__Muribaculaceae (7%) (Figure 8B).

**TABLE 4:** Statistical analysis of reads in different taxa.

Sample	Kingdom	Phylum	Class	Order	Family	Genus	Species
CC1	75,261	75,071	75,065	75,017	73,860	50,526	3,060
CC2	78,503	78,481	78,481	75,954	75,584	59,422	21,274
CC3	79,042	78,171	77,975	77,567	76,968	69,792	4,558
CC4	76,730	74,151	74,087	70,185	68,418	62,388	21,170
CC5	79,000	77,285	77,116	76,573	74,843	62,087	9,946
CC6	79,231	79,207	79,207	76,259	75,909	53,918	10,070
CL1	79,534	79,518	79,518	78,718	78,711	78,335	1,631
CL2	78,409	77,824	77,656	77,138	75,770	63,363	5,955
CL3	77,559	77,544	77,537	74,000	73,939	55,710	11,246
CL4	77,148	77,095	77,093	76,728	76,137	58,235	9,729
CL5	48,233	48,076	48,072	48,023	47,866	43,122	7,841
CL6	74,338	74,165	74,158	72,903	72,781	65,317	13,959
MC1	69,208	68,879	68,877	68,188	66,961	45,445	4,970
MC2	49,218	49,120	49,099	48,978	48,718	31,895	1,332
MC3	79,011	77,206	77,199	75,244	73,489	50,675	6,446
MC4	58,545	58,506	58,499	58,410	58,203	35,689	13,450
MC5	56,852	56,633	56,604	51,276	50,820	33,578	5,152
MC6	79,300	76,213	76,179	69,964	69,301	65,916	21,261
ML1	68,498	68,182	68,179	67,834	67,615	63,701	3,528
ML2	79,092	79,038	79,038	78,779	78,204	50,708	12,429
ML3	68,404	68,342	68,339	68,109	67,847	57,078	4,400
ML4	71,201	71,136	71,133	70,866	70,771	59,408	4,200
ML5	60,238	60,175	60,175	60,030	59,914	57,902	10,959
ML6	118,175	116,506	116,470	115,923	115,416	113,662	10,614

### Marker bacteria in microbiota of mice among different groups

**FIGURE 9:**
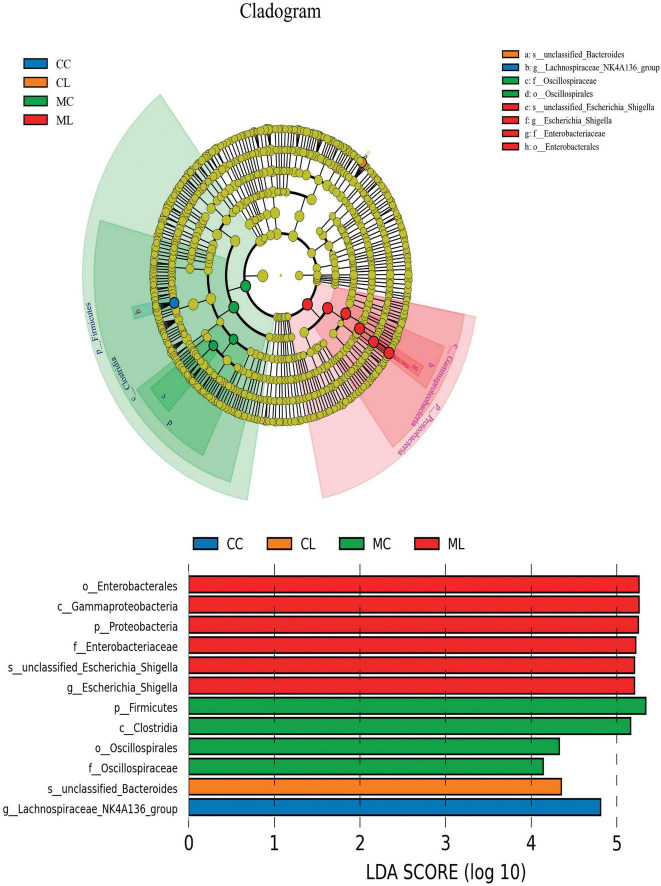
Marker microbiota analysis of mice in different groups via LEfSe analysis.

We first performed LEfSe analysis and found that o__Enterobacterales, C__Gammaproteobacteria, p__Proteobacteria, f__Enterobacteriaceae, s__unclassified_*Escherichia_Shigella*, g__*Escherichia_Shigella*, p__*Firmicutes*, c__*Clostridia*, o__Oscillospirales, f__Oscillospiraceae, s__unclassified_Bacteroides and g__Lachnospiraceae_NK4A136_group were biomakers in mice (Figure 9).

**FIGURE 10:**
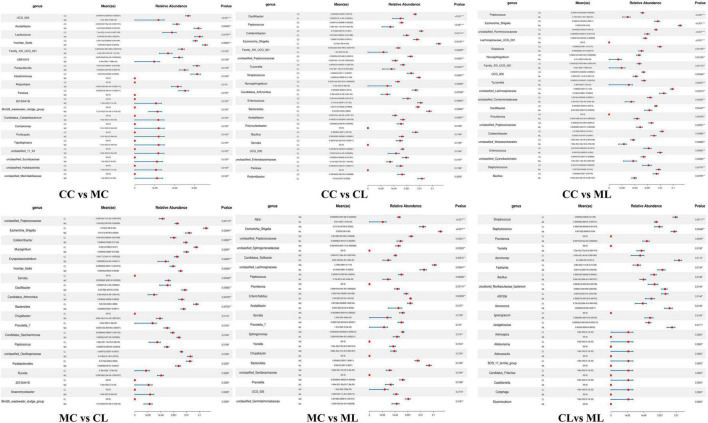
Marker microbiota analysis of mice in different groups via metastats.

Then we used metastats analysis and revealed that compared with CC mice, the abundance of UCG_005 (*P* < 0.001), Family_XIII_001 (*P* < 0.05), UBA1819 (*P* < 0.05), *Parasutterella* (*P* < 0.05), *Intestinimonas* (*P* < 0.05) and Pantoea (*P* < 0.05) were lower in MC mice, while *Acetatifactor* (*P* < 0.01), *Lactococcus* (*P* < 0.01), Incertae_Sedis (*P* < 0.01), Atopostipes (*P* < 0.05), 2013Ark19i (*P* < 0.05), Blvii28_sludge_group (*P* < 0.05), *Candidatus_Caldatribacterium* (*P* < 0.05), *Comamonas* (*P* < 0.05), *Ponticaulis* (*P* < 0.05), Tepidisphaera (*P* < 0.05), unclassified_11_24 (*P* < 0.05), unclassified_Euzebyaceae (*P* < 0.05), unclassified_Halobacterota (*P* < 0.05) and unclassified_Mariniliaceae (*P* < 0.05) were higher. The abundance of *Oscillibacter* (*P* < 0.001), *Peptococcus* (*P* < 0.001), *Colidextribacter* (*P* < 0.01), Family_XIII_001 (*P* < 0.01), unclassified_Peptococcaceae (*P* < 0.01), *Tyzzerella* (*P* < 0.01), *Novosphingobium* (*P* < 0.01), *Candidatus_Arthromitus* (*P* < 0.01), *Polynucleobacter* (*P* < 0.05), *Bacillus* (*P* < 0.05), *Serratia* (*P* < 0.05), UCG_005 (*P* < 0.05), unclassified_Enterobacteriaceae (*P* < 0.05) and Pantoea (*P* < 0.05) were lower in group CL, while *Escherichia_Shigella* (*P* < 0.01), *Streptococcus* (*P* < 0.01), *Enterococcus* (*P* < 0.01), Bacteroides (*P* < 0.01), Acetatifactor (*P* < 0.01) and Rodentibacter (*P* < 0.05) were higher. Compared with mice in group CC, genera of *Peptococcus* (*P* < 0.001), unclassified_Ruminococcaceae (*P* < 0.001), Lachnospiraceae_UCG_001 (*P* < 0.001), *Roseburia* (*P* < 0.01), *Novosphingobium* (*P* < 0.01), Family_XIII_UCG_001 (*P* < 0.01), UCG_005 (*P* < 0.01), *Tyzzerella* (*P* < 0.01), unclassified_Lachnospiraceae (*P* < 0.01), unclassified_Comamonadaceae (*P* < 0.01), *Oscillibacter* (*P* < 0.01), unclassified_Peptococcaceae (*P* < 0.01), *Colidextribacter* (*P* < 0.01), unclassified_Cyanobacteriales (*P* < 0.01) and *Bacillus* (*P* < 0.01) were lower in group ML, while *Escherichia_Shigella* (*P* < 0.001), Providencia (*P* < 0.01), *Enterococcus* (*P* < 0.01) and *Staphylococcus* (*P* < 0.01) were higher. Whereas, compared with MC mice, the abundance of unclassified_Peptococcaceae (*P* < 0.01), *Colidextribacter* (*P* < 0.01), Incertae_Sedis (*P* < 0.01), Serralia (*P* < 0.01), *Oscillibacter* (*P* < 0.01), *Candidatus_Arthromitus* (*P* < 0.01), *Chujaibacter* (*P* < 0.05), *Prevotella*_7 (*P* < 0.05), *Candidatus_Saccharimonas* (*P* < 0.05), *Peptococcus* (*P* < 0.05), unclassified_Oscillospiraceae (*P* < 0.05), *Runella* (*P* < 0.05), 2013Ark19i (*P* < 0.05), *Anaeromyxobacter* (*P* < 0.05) and Blvii28_wastewater_sludge_group (*P* < 0.05) were lower in CL mice, while *Escherichia_Shigella* (*P* < 0.01), *Mucispirillum* (*P* < 0.01), *Erysipelatoclostridium* (*P* < 0.01), Bacteroides (*P* < 0.01), Parabacteroides (*P* < 0.05), were higher. Similarly, compared with mice in group MC, the abundance of RB41 (*P* < 0.001), unclassified_Peptococcaceae (*P* < 0.01), unclassified_Sphingomonadaceae (*P* < 0.01), *Candidatus_Solibacter* (*P* < 0.01), unclassified_Lachnospiraceae (*P* < 0.01), *Peptococcus* (*P* < 0.01), *Acetatifactor* (*P* < 0.05), *Serratia* (*P* < 0.05), *Prevotella*_7 (*P* < 0.05), *Sphingomonas* (*P* < 0.05), *Chujaibacter* (*P* < 0.05), unclassified_Sphingomonadaceae (*P* < 0.05), *Prevotella* (*P* < 0.05) and unclassified_Gemmatimonadaceae (*P* < 0.05) were lower in group ML, while *Escherichia_Shigella* (*P* < 0.001), *Providencia* (*P* < 0.01), *Enterorhabdus* (*P* < 0.01), *Yaniella* (*P* < 0.05), Bacteroides (*P* < 0.05) and UCG_005 (*P* < 0.05) were higher. Compared with mice in CL group, the abundance of Streptococcus (*P* < 0.01), *Bacillus* (*P* < 0.01), ASF356 (*P* < 0.05), Aclinospica (*P* < 0.05), *Aliidiomarina* (*P* < 0.05), *Asticcacaulis* (*P* < 0.05), BC19_17_termte_group (*P* < 0.05), *Candidatus*_Fritschea (*P* < 0.05), *Castellaniella* (*P* < 0.05), Cytophaga (*P* < 0.05) and *Elusimicrobium* (*P* < 0.05) were lower in ML mice, while *Staphylococcus* (*P* < 0.01), *Providencia* (*P* < 0.01), *Yaniella* (*P* < 0.01), *Aeromonas* (*P* < 0.01), Facklamia (*P* < 0.01), uncultured_Muribaculaceae_bacterium (*P* < 0.05), *Aerococcus* (*P* < 0.05), *lgnavigranum* (*P* < 0.05) and *Jeotgalicoccus* (*P* < 0.05) were higher (Figure 10).

**FIGURE 11:**
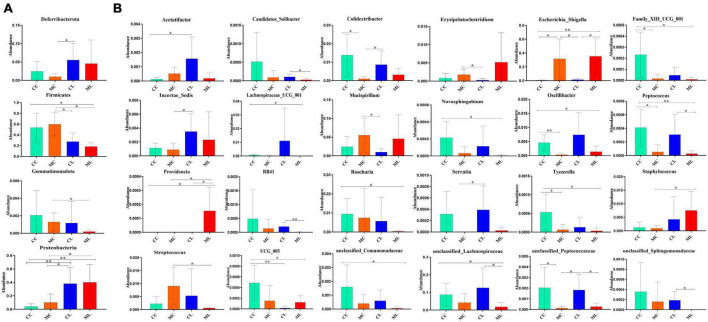
Distinguished microbiota analysis of mice in different groups. **(A)** Phylum, **(B)** Genus. Significance is presented as *p < 0.05, **p < 0.01; data are presented as the mean ± SEM (*n* = 6).

Likewise, we compared the abundance of bacteria among the four groups at the phylum and genus levels. The results showed that the phylum Deferribacterota was significantly higher in CL mice compared to MC animals (*P* < 0.05). Firmicutes was markedly lower in group CL (*P* < 0.05) and ML (*P* < 0.05) compared to group MC, and it was also significantly lower than that in group CC (*P* < 0.05). Gemmatimonadota in ML animals was dramatically lower compared to MC animals (*P* < 0.05). Proteobacteria in CL mice showed a significantly higher abundance compared to CC (*P* < 0.01) and MC (*P* < 0.05) animals, respectively. Similar results were observed in ML mice, with a significantly higher abundance of Proteobacteria in ML compared to CC (*P* < 0.01) and MC (*P* < 0.05) (Figure 11A). At the genus level, the abundance of Acetatifactor in CL mice was significantly higher than in CC animals (*P* < 0.05). *Candidatus_Solibacter* (*P* < 0.05) and unclassified_Sphingomonadaceae (*P* < 0.05) in CL mice were notably higher compared to ML mice. *Colidextribacter* in MC mice was significantly lower than that in CC (*P* < 0.05) and CL (*P* < 0.05) animals. *Erysipelatoclostridium* (*P* < 0.05) and Mucispirillum (*P* < 0.05) in MC animals was markedly higher than that in CL mice. *Escherichia_Shigella* was higher in MC animals than CC (*P* < 0.05) and CL mice (*P* < 0.05). Similarly, this genus was obviously higher found in group ML than groups CC (*P* < 0.01) and CL (*P* < 0.05). Family_XIII_UCG_001 in group CC was higher than group MC (*P* < 0.05) and ML (*P* < 0.05), respectively. Incertae_Sedis (*P* < 0.05) and Serratia (*P* < 0.05) was significantly higher in mice in CL than that in MC. Lachnospiraceae_UCG_001 (*P* < 0.05), *Novosphingobium* (*P* < 0.05), *Roseburia* (*P* < 0.05) and unclassified_Comamonadaceae (*P* < 0.05) in CC mice was notably higher than ML mice, respectively. *Oscillibacter* in CC animals was significantly higher than MC (*P* < 0.01) and ML (*P* < 0.05) groups. *Peptococcus* was discovered higher in group CC than group MC (*P* < 0.05) and ML (*P* < 0.01). Similarly, it was observably higher in CL mice than ML mice (*P* < 0.05). *Providencia* was obviously higher in ML mice than animals in other groups (*P* < 0.05). RB41 was higher in CL groups than ML (*P* < 0.01). *Tyzzerella* in group CC was higher than group MC (*P* < 0.05) and ML (*P* < 0.05). *Staphylococcus* in mice in group ML was significantly higher than it in group MC (*P* < 0.05), while *Streptococcus* in mice in group ML was significantly lower than group MC (*P* < 0.05). UCG_005 in mice in CC was markedly higher than animals in group CL (*P* < 0.01) and ML (*P* < 0.05). The abundance of unclassified_Lachnospiraceae in group ML was significantly lower than it in group CC (*P* < 0.05) and CL (*P* < 0.05). Unclassified_Peptococcaceae in CC animals was markedly higher than MC animals (*P* < 0.05), similarly it was higher in CL mice than MC (*P* < 0.05) and ML (*P* < 0.05) (Figure 11B).

### LPS changed microbiota function in mice among different groups

**FIGURE 12:**
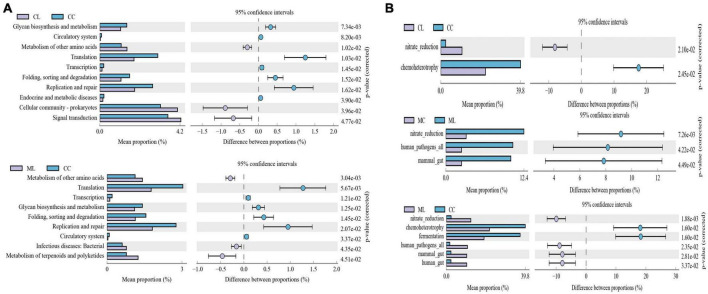
Potential Function prediction analysis of mouse microbiota. **(A)** Tax4Fun, **(B)** FAPROTAX.

Function prediction via Picrust2 showed that obviously different functions of KEGG level of organismal systems (*P* < 0.05), genetic information processing (*P* < 0.05) and metabolism (*P* < 0.05) were examined between CC and ML mice (Table 5). Phenotypic analysis bugbase revealed that Contains_Mobile_Elements (*P* < 0.05), Gram_Negative (*P* < 0.05) and Gram_Positive were observably higher in ML mice, Facultatively_Anaerobic (*P* < 0.05), Potentially_Pathogenic (*P* < 0.05) and Stress_Tolerant (*P* < 0.05) were significantly higher in mice in CL and ML (Table 6). Tax4Fun analysis found that glycan biosynthesis and metabolism (GBD) (*P* < 0.01), circulatory system (*P* < 0.01), translation (*P* < 0.05), transcription (*P* < 0.05), folding, sorting and degradation (FSD) (*P* < 0.05), replication and repair (RR) (*P* < 0.05), and endocrine and metabolic diseases (EMDs) in CL animals were obviously lower than mice in CC, while metabolism of other amino acids (*P* < 0.05), cellular community-prokaryotes (*P* < 0.05) and signal transduction (*P* < 0.05) were significantly higher. Compared with mice in group CC, metabolism of other amino acids (*P* < 0.01), infectious diseases: bacterial (*P* < 0.05) and metabolism of terpenoids and polyketides (*P* < 0.05) were observably higher, while translation (*P* < 0.01), transcription (*P* < 0.05), GBD (*P* < 0.05), FSD (*P* < 0.05), RR (*P* < 0.05), and circulatory system (*P* < 0.05) were lower (Figure 12A). FAPROTAX analysis showed that nitrate reduction (*P* < 0.05) and chemoheterotrophy (*P* < 0.05) in CL and ML were memorably lower than CC, while nitrate reduction (*P* < 0.01), human pathogens all (*P* < 0.05), mammal gut (*P* < 0.05) and human gut (*P* < 0.05) were significantly higher in ML. Compared with animals in MC, nitrate reduction (*P* < 0.01), human pathogens (*P* < 0.05) and mammal gut (*P* < 0.05) were obviously higher in animals in ML group (Figure 12B).

**TABLE 5:** Comparing KEGG level 1 function of mice microbiota in different groups via picrust2.

Class 1	CC	MC	CL	ML
Organismal systems	1.29 ± 0.06	1.30 ± 0.04	1.35 ± 0.04	1.37 ± 0.05*
Cellular processes	3.73 ± 0.39	3.68 ± 0.61	3.44 ± 0.18	3.53 ± 0.24
Human diseases	2.77 ± 0.40	2.73 ± 0.25	2.96 ± 0.26	3.04 ± 0.22
Genetic information processing	8.84 ± 0.87	8.83 ± 1.34	7.93 ± 0.65	7.60 ± 0.63*
Environmental information processing	7.27 ± 0.83	7.45 ± 0.0.40	7.52 ± 0.86	7.33 ± 0.82
Metabolism	76.10 ± 0.63	76.00 ± 0.81	76.80 ± 0.97	77.13 ± 0.57*

Data are presented as the mean ± std.dev (*n* = 6), significance is presented as *p < 0.05.

**TABLE 6:** Comparing phenotypic analysis of mice microbiota in different groups via bugbase prediction.

Phenotypes	CC	MC	CL	ML
Aerobic	37.76 ± 29.96	31.33 ± 14.09	21.06 ± 11.56	26.67 ± 20.41
Anaerobic	52.61 ± 33.35	59.77 ± 22.80	44.30 ± 20.85	38.31 ± 24.41
Contains_Mobile_Elements	0.32 ± 0.34^a^	1.58 ± 2.14^a^	23.67 ± 24.27^a^	23.04 ± 15.89^b^
Facultatively_Anaerobic	7.70 ± 9.35^a^	7.53 ± 9.23^a^	32.57 ± 20.47^b^	33.28 ± 21.12^b^
Forms_Biofilms	0.64 ± 0.70	0.42 ± 0.55	4.95 ± 7.71	0.94 ± 0.93
Gram_Negative	46.75 ± 27.30^a^	44.81 ± 26.25^a^	69.34 ± 16.51^a^	76.68 ± 8.18^b^
Gram_Positive	53.25 ± 27.30^a^	55.19 ± 26.25^a^	30.66 ± 16.51^a^	23.32 ± 8.18^b^
Potentially_Pathogenic	0.87 ± 0.86^a^	1.71 ± 2.14^a^	27.73 ± 21.69^b^	23.14 ± 15.91^b^
Stress_Tolerant	1.05 ± 1.05^a^	3.52 ± 3.67^a^	29.99 ± 20.34^b^	25.32 ± 16.90^b^

Data are presented as the mean ± std.dev (*n* = 6), significance is presented as different letters when *p* < 0.05.

### LPS and cold normal saline changed metabolites in mice among different groups

**FIGURE 13:**
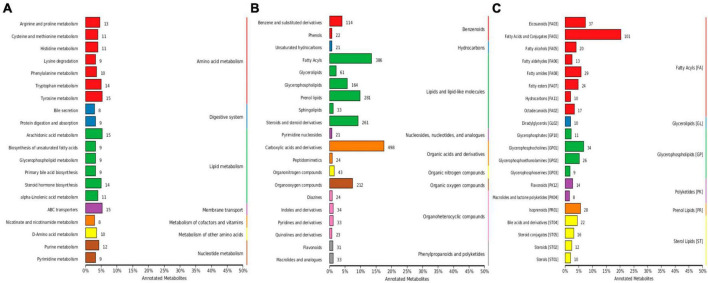
Metabolites annotation of mice. **(A)** KEGG, **(B)** HMDB, and **(C)** LIPID MAPS.

**FIGURE 14:**
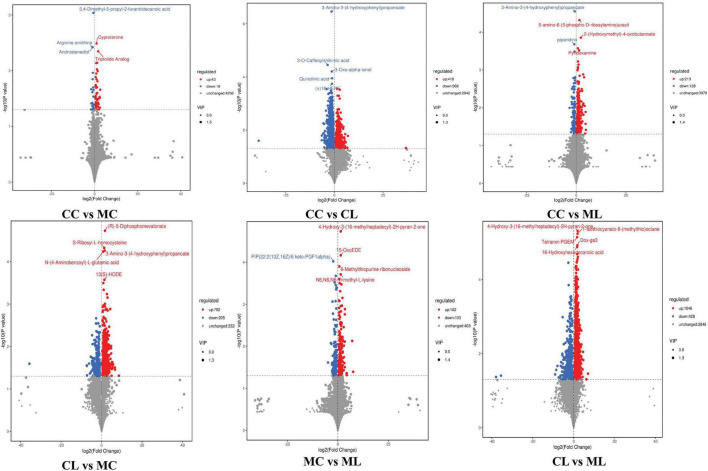
Volcano plot of differential metabolites in mice.

**FIGURE 15:**
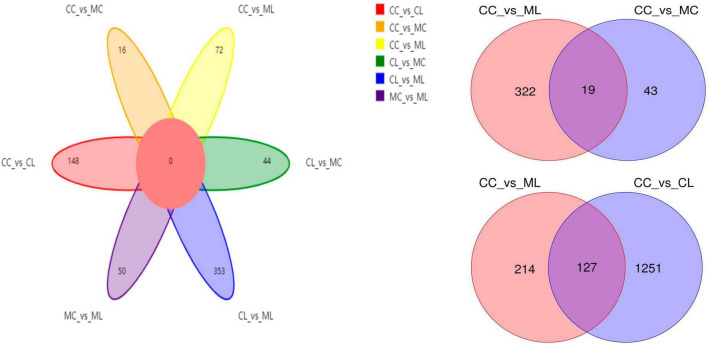
Venn map of differential metabolites among different mice groups.

Out of 4,320 metabolites, 2,181 and 2,139 were in positive and negative ion mode, respectively in mice in our study. Those metabolites were mainly annotated to amino acid metabolism and lipid metabolism via KEGG (Figure 13A), lipids and lipid-like molecules and organic acids and derivatives via HMDB (Figure 13B), and fatty acyls and glycerolipids via LIPID MAPS (Figure 13C). Compared with CC mice, there were 43 up-regulated and 19 down-regulated metabolites in MC animal, 410 up-regulated and 968 down-regulated metabolites in CL animal, while 213 up-regulated and 128 down-regulated metabolites in CL animals. Compared with group MC, there were 205 up-regulated and 792 down-regulated metabolites in CL animal, whereas 182 up-regulated and 103 down-regulated metabolites in ML animals. Compared with CL group, there were 1,046 up-regulated and 428 down-regulated metabolites in ML animals (Figure 14). Venn map showed that there were no shared differential metabolites among mice groups (Figure 15). Z-score analysis of top 30 differential metabolites in animals showed that the abundance of metabolites in mice treated with ice-cold normal saline decreased with red balls mainly distributed in MC and ML sides, while LPS challenging could increase the abundance of metabolites with more red balls in the CL sides (Supplementary Figure 2). To further reveal the marker metabolites between mice groups, we examined metabolites between CC vs. MC and CC vs. ML. The results showed that compared with mice in group CC, neg_3481 (*P* < 0.05), neg_457 (*P* < 0.01), neg_7126 (*P* < 0.05), pos_771 (*P* < 0.05), pos_715 (*P* < 0.05), neg_539 (*P* < 0.05), neg_4796 (*P* < 0.05), pos_1504 (*P* < 0.05), pos_3391 (*P* < 0.05), neg_6883 (*P* < 0.05) and pos_4916 (*P* < 0.05) were obviously higher in group MC and ML, while neg_6324 (*P* < 0.05) and pos_783 (*P* < 0.05) were significantly lower in group MC and ML. The abundance of neg_6169 (*P* < 0.05) and neg_6271 (*P* < 0.05) were observably lower in MC than CC, while higher in ML. The abundance of neg_1751 (*P* < 0.01), neg_87 (*P* < 0.01), pos_699 (*P* < 0.05) and pos_4607 (*P* < 0.05) in MC were markedly higher (*P* < 0.05) than CC, while lower in ML (Supplementary Figure 3). Whereas, comparing of metabolites in mice in CC indicated that 127 shared differential metabolites in group CL and ML, with 94 lower abundant metabolites and 33 higher abundant metabolites (Table 7).

**TABLE 7:** Comparing of differential metabolites among different mice groups.

ID	CC	CL	ML
neg_1198	33.07 ± 17.46^a^	135.20 ± 74.40^b^	72.99 ± 28.02^b^
neg_1556	3836.96 ± 315.62^b^	2990.18 ± 622.50^a^	2987.65 ± 661.52^a^
neg_1598	6562.27 ± 3649.38^b^	1409.85 ± 958.93^a^	1897.95 ± 959.68^a^
neg_1683	164.84 ± 87.20^b^	35.87 ± 23.49^a^	31.38 ± 21.59^a^
neg_1745	127.60 ± 27.63^c^	50.20 ± 27.30^a^	82.88 ± 13.63^b^
neg_1751	27.41 ± 12.37^b^	8.13 ± 7.14^a^	9.98 ± 7.01^a^
neg_1790	213.76 ± 19.45^c^	75.04 ± 17.08^a^	126.06 ± 10.16^b^
neg_1819	33.69 ± 31.76^a^	82.37 ± 26.70^b^	217.30 ± 142.44^b^
neg_1830	758.31 ± 184.44^b^	391.08 ± 172.60^a^	396.48 ± 122.24^a^
neg_2058	11.35 ± 9.14^a^	28.81 ± 10.51^b^	40.49 ± 22.07^b^
neg_2088	6140.61 ± 2537.07^b^	2008.73 ± 470.76^a^	2125.20 ± 798.61^a^
neg_2352	754.67 ± 1084.69^a^	3595.94 ± 1739.60^b^	3417.12 ± 2193.03^b^
neg_3041	161.69 ± 62.16^b^	77.94 ± 39.70^a^	241.30 ± 46.43^c^
neg_3305	738.85 ± 111.02^b^	458.96 ± 129.22^a^	462.15 ± 96.80^a^
neg_335	194.08 ± 29.05^b^	86.22 ± 26.31^a^	120.24 ± 25.24^a^
neg_336	273.50 ± 108.99^b^	44.32 ± 25.33^a^	117.69 ± 79.59^a^
neg_3439	105.94 ± 140.46^a^	493.43 ± 238.22^b^	320.37 ± 124.28^b^
neg_3667	4.79 ± 9.70^a^	68.50 ± 48.74^b^	71.84 ± 26.57^b^
neg_3765	1514.62 ± 509.87^b^	530.18 ± 192.89^a^	812.15 ± 258.59^a^
neg_3801	24414.46 ± 8231.70^b^	9737.90 ± 5857.18^a^	12475.74 ± 5106.68^a^
neg_381	520.97 ± 131.36^c^	89.03 ± 19.11^a^	218.78 ± 90.63^b^
neg_3954	23780.12 ± 7905.96^c^	8040.20 ± 4143.39^a^	13795.74 ± 2672.11^b^
neg_406	17735.61 ± 5578.39^c^	2080.01 ± 339.00^a^	7906.46 ± 3804.20^b^
neg_4452	6984.31 ± 2014.20^b^	4174.15 ± 1406.84^a^	4244.05 ± 582.62^a^
neg_471	296.71 ± 69.52^b^	167.78 ± 41.54^a^	211.55 ± 44.99^a^
neg_4774	1014.78 ± 378.38^c^	195.90 ± 143.81^a^	563.30 ± 209.31^b^
neg_478	884.96 ± 264.79^c^	286.06 ± 71.49^a^	547.49 ± 176.05^b^
neg_4796	517.57 ± 192.50^a^	1256.56 ± 602.77^b^	1693.47 ± 746.30^b^
neg_485	8061.86 ± 2603.25^c^	730.48 ± 561.39^a^	4198.18 ± 2243.77^b^
neg_4878	2368.18 ± 856.82^c^	497.93 ± 208.63^a^	1153.53 ± 402.62^b^
neg_4939	14443.58 ± 3996.24^c^	3704.55 ± 1851.07^a^	6653.47 ± 2168.27^b^
neg_4970	169.74 ± 43.30^a^	1131.30 ± 668.86^b^	2004.70 ± 954.91^b^
neg_508	1019.59 ± 261.38^b^	513.35 ± 180.02^a^	628.64 ± 184.37^a^
neg_5089	2002.32 ± 1269.18^a^	6585.82 ± 3665.93^b^	10566.29 ± 5215.02^b^
neg_5210	4117.40 ± 1611.32^c^	666.28 ± 314.09^a^	1701.79 ± 707.80^b^
neg_5222	158435.10 ± 46708.06^c^	45323.48 ± 21375.06^a^	96036.72 ± 28872.87^b^
neg_5224	371.72 ± 126.98^b^	121.36 ± 73.08^a^	190.93 ± 86.04^a^
neg_5253	547.70 ± 124.64^c^	163.79 ± 76.62^a^	342.35 ± 153.65^b^
neg_5329	448.83 ± 70.37^b^	299.41 ± 91.80^a^	339.57 ± 79.46^a^
neg_5399	31.22 ± 29.09^a^	127.88 ± 80.19^b^	571.94 ± 292.91^c^
neg_5406	11726.23 ± 4434.50^b^	3089.03 ± 798.86^a^	4195.31 ± 742.53^c^
neg_5572	20.51 ± 18.20^a^	83.47 ± 30.47^b^	112.16 ± 70.99^b^
neg_5592	2307.16 ± 1094.87^a^	4817.65 ± 1244.49^b^	4163.16 ± 874.03^b^
neg_574	4833.78 ± 2092.78^c^	572.14 ± 95.36^a^	1962.32 ± 1287.23^b^
neg_577	2607.41 ± 1131.95^b^	331.70 ± 158.62^a^	650.89 ± 428.41^a^
neg_578	492.53 ± 131.40^b^	146.92 ± 56.56^a^	236.18 ± 122.00^a^
neg_581	2669.57 ± 1179.95^b^	467.98 ± 76.42^a^	1103.83 ± 713.88^a^
neg_601	494.69 ± 120.96^b^	155.81 ± 62.66^a^	283.54 ± 148.32^a^
neg_6173	291.47 ± 62.71^a^	549.08 ± 158.00^b^	743.58 ± 187.45^b^
neg_6221	21.03 ± 13.82^a^	63.75 ± 12.91^b^	106.48 ± 60.37^b^
neg_6242	1423.83 ± 674.37^a^	11954.68 ± 8368.04^b^	3864.34 ± 2024.83^b^
neg_6324	4399.15 ± 1060.33^b^	1688.54 ± 788.53^a^	2732.95 ± 963.99^a^
neg_6362	1253.77 ± 257.68^b^	555.29 ± 238.83^a^	690.29 ± 268.98^a^
neg_6432	2462.59 ± 572.13^b^	1117.27 ± 492.70^a^	1325.89 ± 533.48^a^
neg_6522	2584.38 ± 1399.82^b^	888.98 ± 791.94^a^	695.70 ± 300.39^a^
neg_6581	13.00 ± 11.83^a^	167.00 ± 100.66^b^	64.13 ± 33.06^b^
neg_6582	892.06 ± 701.20^a^	7449.60 ± 4601.59^b^	3956.39 ± 2164.10^b^
neg_671	26968.05 ± 13838.66^c^	2798.41 ± 782.76^a^	8661.32 ± 5462.16^b^
neg_674	4851.40 ± 1084.60^b^	2278.15 ± 482.32^a^	2862.09 ± 853.62^a^
neg_6765	21.76 ± 27.96^a^	299.10 ± 219.90^b^	479.13 ± 393.34^b^
neg_6823	1346.85 ± 758.51^a^	4833.82 ± 2452.01^b^	3830.03 ± 1958.09^b^
neg_6913	137886.28 ± 97522.72^b^	11193.54 ± 7971.42^a^	21812.49 ± 28529.04^a^
neg_7013	6143.66 ± 906.40^a^	53349.97 ± 24038.65^b^	39412.50 ± 25899.05^b^
neg_830	63.66 ± 13.48^b^	44.98 ± 5.54^a^	41.65 ± 8.49^a^
neg_836	19475.29 ± 8469.42^b^	1143.80 ± 568.40^a^	1143.80 ± 568.40^a^
neg_85	25.29 ± 17.46^a^	74.76 ± 24.70^b^	57.46 ± 8.44^b^
neg_88	131.02 ± 30.28^b^	38.22 ± 24.16^a^	64.16 ± 23.64^a^
neg_928	4758.94 ± 1151.76^b^	2315.88 ± 396.22^a^	2618.04 ± 450.70^a^
neg_929	3861.78 ± 1502.59^b^	657.04 ± 152.17^a^	785.12 ± 370.81^a^
neg_934	375.91 ± 87.21^b^	244.99 ± 46.84^a^	223.38 ± 46.87^a^
neg_962	2387.74 ± 872.87^b^	347.79 ± 158.42^a^	691.30 ± 332.90^a^
pos_101	35.26 ± 13.70^b^	9.01 ± 9.04^a^	16.66 ± 11.67^a^
pos_111	179.65 ± 33.57^b^	127.09 ± 38.49^a^	270.18 ± 41.82^c^
pos_120	162.89 ± 28.43^b^	104.68 ± 42.31^a^	226.26 ± 27.28^c^
pos_1241	1612.74 ± 384.75^b^	639.20 ± 277.29^a^	667.70 ± 589.61^a^
pos_135	21.76 ± 8.64^b^	6.16 ± 4.75^a^	8.53 ± 4.91^a^
pos_137	55.49 ± 14.07^b^	34.73 ± 14.12^a^	75.85 ± 13.45^c^
pos_1527	1433.16 ± 369.76^b^	960.75 ± 187.49^a^	897.95 ± 267.67^a^
pos_1678	3732.37 ± 2687.22^a^	9030.32 ± 3598.49^b^	9951.02 ± 4849.47^b^
pos_190	1270.08 ± 274.45^b^	478.72 ± 268.87^a^	661.93 ± 181.21^a^
pos_305	28.68 ± 17.86^b^	4.67 ± 5.39^a^	62.10 ± 23.63^c^
pos_318	1515.25 ± 645.48^b^	146.11 ± 73.63^a^	305.24 ± 210.36^a^
pos_3262	523.14 ± 225.11^c^	59.23 ± 21.70^a^	158.94 ± 85.34^b^
pos_3412	176.14 ± 70.73^c^	25.76 ± 10.59^a^	86.33 ± 43.74^b^
pos_355	83.47 ± 35.41^c^	6.09 ± 6.91^a^	40.96 ± 15.82^b^
pos_4053	183.61 ± 91.59^a^	394.88 ± 64.95^b^	448.39 ± 186.59^b^
pos_461	45.02 ± 5.04^b^	24.97 ± 16.66^a^	22.40 ± 6.90^a^
pos_4633	588.10 ± 152.55^b^	290.05 ± 142.60^a^	221.15 ± 82.51^a^
pos_482	776.77 ± 369.55^c^	19.85 ± 22.17^a^	298.62 ± 243.72^b^
pos_498	600.32 ± 275.63^b^	146.84 ± 71.72^a^	260.20 ± 174.62^a^
pos_5000	2707.16 ± 1052.65^b^	1434.77 ± 553.10^a^	4528.64 ± 1449.18^c^
pos_5165	243.17 ± 122.32^a^	705.73 ± 214.14^b^	2681.04 ± 861.58^c^
pos_5199	151.46 ± 88.02^a^	275.60 ± 84.50^b^	596.29 ± 252.17^c^
pos_5239	25.00 ± 37.13^a^	146.34 ± 86.50^b^	114.10 ± 52.48^b^
pos_5265	304.55 ± 102.30^c^	106.81 ± 33.47^a^	166.58 ± 41.40^b^
pos_5266	146.16 ± 47.13^b^	57.87 ± 15.77^a^	78.94 ± 17.50^a^
pos_5268	97.19 ± 26.85^b^	37.01 ± 11.62^a^	41.00 ± 18.13^a^
pos_5269	2801.55 ± 1037.16^b^	1304.27 ± 459.16^a^	1537.39 ± 439.94^a^
pos_5424	1729.51 ± 604.11^b^	604.74 ± 392.17^a^	886.82 ± 409.98^a^
pos_5531	948.73 ± 281.52^b^	516.96 ± 261.90^a^	435.77 ± 95.40^a^
pos_5899	542.57 ± 196.19^a^	3892.82 ± 2543.66^b^	3370.83 ± 2021.23^b^
pos_598	140361.16 ± 43082.17^a^	24809.18 ± 14315.74^b^	24809.18 ± 14315.74^b^
pos_6025	185.22 ± 47.41^b^	105.43 ± 44.50^a^	118.67 ± 37.25^a^
pos_6064	733.73 ± 209.51^a^	1235.90 ± 230.23^b^	2146.20 ± 762.14^c^
pos_609	195.47 ± 97.71^c^	14.30 ± 10.57^a^	72.67 ± 43.36^b^
pos_6099	1577.45 ± 454.01^a^	15237.29 ± 11320.29^c^	12216.60 ± 4648.55^b^
pos_6146	6115.40 ± 2305.52^c^	1324.47 ± 415.27^a^	3094.72 ± 536.07^b^
pos_619	5394.04 ± 1664.79^b^	1136.21 ± 756.32^a^	2773.09 ± 1707.43^a^
pos_620	39.25 ± 12.09^b^	8.36 ± 4.46^a^	13.10 ± 12.97^a^
pos_621	773.71 ± 247.55^b^	129.33 ± 69.45^a^	309.73 ± 232.69^a^
pos_6249	2768.17 ± 778.71^c^	877.96 ± 414.92^a^	1831.54 ± 423.24^b^
pos_6405	809.69 ± 229.70^a^	1882.51 ± 609.71^b^	1635.54 ± 407.97^b^
pos_6646	1872.51 ± 694.98^b^	708.78 ± 365.46^a^	877.43 ± 198.16^a^
pos_6725	276.60 ± 76.67^b^	128.63 ± 97.98^a^	155.60 ± 57.67^a^
pos_6889	10388.01 ± 2876.39^c^	2620.05 ± 1556.63^a^	5667.42 ± 2170.47^b^
pos_6961	248.79 ± 97.16^b^	89.13 ± 49.15^a^	425.01 ± 141.92^c^
pos_7617	519.30 ± 84.38^a^	2461.45 ± 1081.21^b^	2790.60 ± 1801.86^b^
pos_762	179.67 ± 55.39^b^	61.29 ± 24.87^a^	52.46 ± 45.29^a^
pos_7698	13132.79 ± 4950.31^a^	21968.14 ± 6396.33^b^	34126.43 ± 10518.70^b^
pos_7749	584.61 ± 112.35^b^	392.79 ± 137.82^a^	911.55 ± 72.67^c^
pos_783	227.54 ± 90.95^b^	33.10 ± 28.85^a^	94.55 ± 72.67^a^
pos_8045	685.39 ± 148.84^b^	406.45 ± 103.18^a^	886.37 ± 115.58^c^
pos_806	243.82 ± 118.45^b^	23.22 ± 28.23^a^	69.21 ± 114.87^a^
pos_8108	540.46 ± 117.12^b^	314.67 ± 116.01^a^	784.02 ± 94.97^c^
pos_8112	7987.51 ± 1358.04^b^	5198.31 ± 1640.70^a^	10436.67 ± 1341.77^c^
pos_832	9894.85 ± 3939.77^b^	1387.88 ± 561.12^a^	3719.36 ± 3176.71^a^
pos_99	79.63 ± 16.18^b^	30.53 ± 19.93^a^	35.69 ± 19.49^a^

Data are presented as the mean ± std.dev (*n* = 6), significance is presented as different letters when *p* < 0.05.

### Correlation analysis of gut microbiota and inflammatory cytokines in mice

Correlation analysis showed that *Enterococcus* and *Escherichia_Shigella* were both positively related to TNF-α, IL-1β and IL-6, while Lachnospiraceae_UCG_001 and unclassified_Lachnospiraceae were negatively related to these inflammatory cytokines. Bacteroides was positively related to TNF-α, Parateroides was positively related to IL-10, TNF-α and IL-1β, and unclassified_Muribaculaceae was positively related to IL-10, while *Colidextribacter* and Lachnospiraceae_NK4A136_group were negatively related to IL-1β and IL-6 (Supplementary Figure 4).

## Discussion

During the winter period or in cold areas, cold water can induce stress in animals. When it is combined with factors such as feed supplements, social factors and environmental stresses, it can significantly impair the function of the digestive system in animals, and lead to growth performance disorders (Yin et al., 2014; Oraby et al., 2021; Rehman et al., 2021). In this study, we aimed to investigate the impact of cold stimulus combined with LPS on mice. Our findings revealed that the mice treated with cold normal saline exhibited a slightly lower body weight. This observation is consistent with previous studies conducted on pigs in which different temperature water treatments influenced the body weight (Zhang et al., 2020). Pathological analysis revealed that cold stress had minimal impact on the integrity of intestinal villi and gastric epithelium, while LPS significantly destroyed the villi. These findings are congrant with previous studies conducted on cold-stressed broilers (Su et al., 2018) and LPS-challenged hens (Feng et al., 2023).

Furthermore, the cytokines TNF-α and IL-6 were significantly more expressed in mice challenged with LPS and cold normal saline stress compared to other groups. Whereas, IL-1β level was similar between groups CC and CL, but it was significantly elevated in mice treated with cold normal saline + LPS. Previous study reported that chronic cold exposure upregulated IL6 and TNFα level in the blood of mice (Bal et al., 2017). Our findings are in line with their results in which cold normal saline stress increase the expression of TNF-α and IL-6.

To explore the potential mechanisms, we detected the gene expressions of tight junction proteins in small intestine (jejunum and ileum). Among them OCCLUDIN is recognized as important component of intestinal permeability (Chen et al., 2015, 2022). Whereas, relative gene expression confirmed that significant differences were detected in the expression levels of *OCCLUDIN* and *CASPASE-1* in mice in the CL and ML groups. The expression of *OCCLUDIN* is in line with a study on inflammatory bowel disease in humans (Chen et al., 2015), while expression of *CLAUDIN* is in line with He et al. (2022). Additionally, slight differences in *CLAUDIN* and *NLRP3* were observed between mice in the CL and ML groups. Previous studies found that the activation of *Caspase-1* by *NLRP3* cause inflammation reaction (Sho and Xu, 2019; He et al., 2022).

Moreover, our study assessed the antioxidant indexes, NO levels, and cytokine levels in the serum of various groups in mice. Our findings revealed that cold stress reduced the antioxidant capacity in LPS-challenged mice by lowering the level of T-AOC, GSH-Px, and SOD, and increasing the level of MDA. Additionally, cold stress promoted an inflammatory response, as evidenced by higher levels of IL-1β in mice treated with cold normal saline + LPS. Previous studies have mentioned that the expression of antioxidant is disturbed in different inflammatory conditions (Sho and Xu, 2019; Aziz et al., 2021; Murtaza et al., 2021). Gut microbiome analyzing showed that cold stress led to a decrease in data numbers in the MC group, as well as a reduction in the Shannon and Simpson indexes in the ML group. Moreover, cold stress increased the beta diversities of PCA, PCoA, and NMDS.

To further investigate the distinguished bacteria influenced by cold stress and LPS, we conducted LEfSe analysis and identified 12 biomarkers (o__Enterobacterales, C__Gammaproteobacteria, p__Proteobacteria, f__Enterobacteriaceae, s__unclassified_*Escherichia_Shigella*, g__*Escherichia_Shigella*, p__Firmicutes, c__*Clostridia*, o__Oscillospirales, f__Oscillospiraceae, s__unclassified_Bacteroides and g__Lachnospiraceae_NK4A136_group) in different mouse groups, which was partly in line with the results in cold stress treated rates (Sun et al., 2023). Among them higher abundance of pathogenic s__unclassified_*Escherichia_Shigella* and g__*Escherichia_Shigella* were found in mice in ML, which inferred that cold stress could promote the colonization of harmful bacteria in LPS induced mice. When compared with CC mice, the abundance of 20, 20, and 19 genera were obviously different with MC, CL, and MC animals, respectively. Compared with MC mice, the abundance of 20 and 20 genus were prominently different with mice in CL and ML groups, respectively. There were different 20 genus between CL and ML. Further analysis revealed significant differences in the abundance of 4 phyla and 24 genera were among the mouse groups. Notably, the abundance of *Candidatus_Solibacter* in the ML group was lower compared to the other groups, particularly the CL group. Previous studies have reported a positive correlation between *Candidatus Solibacter*, *Peptococcus*, and antioxidant capacity (Peng et al., 2021; Kong et al., 2022), which is suggesting that the decreased abundance of these genera in the MC and ML groups may indicate reduced oxidative resistance in animals exposed to cold stress.

Additionally, *Escherichia_Shigella* is a genus known to cause mucosal inflammation and has been found in abundant in mice with ulcerative colitis and individuals with Crohn’s disease (Jialing et al., 2020; Ma et al., 2022). The higher abundance of this genus observed in mice in the MC and ML groups, particularly in the ML animals, is consistent with previous studies (Chen et al., 2022). This finding suggests that cold stress may exacerbate intestinal inflammation in mice. On the other hand, lower abundances of Family_XIII_UCG_001, Lachnospiraceae_UCG_001, *Novosphingobium*, RB41, and *Tyzzerella* have been previously reported in chronic colitis mice (Huangfu et al., 2021; Xu et al., 2021), Crohn’s disease patients (Jiang et al., 2022), ulcerative colitis mice (Wang et al., 2019), heat stress-induced rabbits (Shi et al., 2022) and Alzheimer patients (Kaiyrlykyzy et al., 2022), respectively. These findings are consistent with the observations in the MC and ML groups of the current study, which indicated that cold stress may have a negative impact on mice by reducing the abundance of these four genera.

The proportion of Mucispirillum was higher in cold stressed mice, which was in agreement with findings in colitis mouse (Li et al., 2022). Providencia is an opportunistic pathogenic genera known to cause acute enteric infection (Ovchinnikova et al., 2013), and its higher abundance has been previously reported in diarrheal dogs (Herstad et al., 2021). The increased abundance of this pathogenic genus may contribute to intestinal injury in mice. *Staphylococcus* is also a pathogenic genera threatening public health (Hoveida et al., 2019), which may infer that this genera negatively affect animals in the current study.

Previous studies found that unclassified_Lachnospiraceae, Unclassified_Peptococcaceae and unclassified_Sphingomonadaceae were negatively associated with the pathogenesis of type 2 diabetes (Kim et al., 2022), pulmonary fibrosis (Li et al., 2022), and mastitis in camel, respectively. These findings are consistent with the decreased abundance of these three genera observed in the cold-stressed animals in the current study. *Roseburia* is a promising probiotic genus known to improve the gut ecosystem (Sanders et al., 2019; Seo et al., 2020). The lower abundance of *Roseburia* in the MC and ML groups may indicate that cold stress contributes to damage by reducing the presence of this genus.

Cold stress and LPS induction also had an impact on the metabolites in mice. We detected a total of 4,320 metabolites, with 43 up-regulated and 19 down-regulated metabolites in the CC vs. MC animal comparison. Similarly, in the comparison of ML vs. CL animals, we observed 1,046 up-regulated and 428 down-regulated metabolites. Z-score analysis further confirmed the changes in metabolites induced by cold stress. Among these metabolites, there were 19 that showed significant changes between CC vs. MC and CC vs. ML groups. These metabolites include (neg_3481, neg_457, neg_7126, pos_771, pos_715, neg_539, neg_4796, pos_1504, pos_3391, neg_6883, pos_4916, neg_6324, pos_783, neg_6169, neg_6271, neg_1751, neg_87, pos_699, and pos_4607). The alterations in these metabolites, induced by cold stress and LPS, ultimately led to changes in microbiota function.

There are many reports in which it is mentioned that systemic LPS treatment in mice severely impact whole body temperature as well as induce thermogenesis proteins in the skeletal muscle (Bal et al., 2021). In our study, LPS treatment is probably is not systemic, that’s why there were no noticeable induction of thermogenesis proteins in the skeletal muscle. This may be a limitation of present study.

## Conclusion

In conclusion, we investigated the impact of cold stress on LPS-induced mice and observed that cold stress exacerbated intestinal damage by disrupting the balance of gut microbiota and altering its metabolites. These findings have important implications for improving the feeding and management practices of livestock in cold regions or during cold periods.

## Data availability statement

The data presented in this study are deposited in the NCBI database under BioProject accession number PRJNA972973 (https://www.ncbi.nlm.nih.gov/bioproject/PRJNA972973/).

## Ethics statement

All the experiment operations were under the instructions and approval of Laboratory Animals Research Centre of Jiangsu, China and the Ethics Committee of Nanjing Agricultural University. The study was conducted in accordance with the local legislation and institutional requirements.

## Author contributions

JL: Data curation, Formal analysis, Investigation, Methodology, Writing—original draft, Writing—review and editing. ZC: Data curation, Investigation, Methodology, Formal analysis, Writing—review and editing. MW: Data curation, Investigation, Methodology, Writing—review and editing. MA: Formal analysis, Validation, Writing—review and editing. PY: Conceptualization, Funding acquisition, Project administration, Supervision, Validation, Visualization, Writing—review and editing.

## Supplementary material

Supplementary Figure 1 Comparing of villus height and crypt depth in mice in different groups. **(A)** jejunum, **(B)** ileum, **(C)** colon. Significance is presented as **p* < 0.05, ***p* < 0.01, ****p* < 0.001, and *****p* < 0.0001; data are presented as the mean ± SEM (*n* = 3).

Supplementary Figure 2 Z-score analysis of top 30 differential metabolites among different mice groups.

Supplementary Figure 3 Comparing of differential metabolites among different mice groups.

Supplementary Figure 4 Analysis of the correlation between gut microbiota and inflammatory cytokines in mice.
